# LoRa Communications as an Enabler for Internet of Drones towards Large-Scale Livestock Monitoring in Rural Farms

**DOI:** 10.3390/s21155044

**Published:** 2021-07-26

**Authors:** Mehran Behjati, Aishah Binti Mohd Noh, Haider A. H. Alobaidy, Muhammad Aidiel Zulkifley, Rosdiadee Nordin, Nor Fadzilah Abdullah

**Affiliations:** Department of Electrical, Electronics and Systems Engineering, Faculty of Engineering and Built Environment, Universiti Kebangsaan Malaysia, Bangi 43600, Selangor, Malaysia; mehran.behjati@ukm.edu.my (M.B.); aishahmohdnoh94@gmail.com (A.B.M.N.); P92976@siswa.ukm.edu.my (H.A.H.A.); A159788@siswa.ukm.edu.my (M.A.Z.); fadzilah.abdullah@ukm.edu.my (N.F.A.)

**Keywords:** unmanned aircraft vehicle (UAV), drone, long range (LoRa), wireless sensor network, Internet of Things (IoT), remote sensing, smart farming, path planning

## Abstract

Currently, smart farming is considered an effective solution to enhance the productivity of farms; thereby, it has recently received broad interest from service providers to offer a wide range of applications, from pest identification to asset monitoring. Although the emergence of digital technologies, such as the Internet of Things (IoT) and low-power wide-area networks (LPWANs), has led to significant advances in the smart farming industry, farming operations still need more efficient solutions. On the other hand, the utilization of unmanned aerial vehicles (UAVs), also known as drones, is growing rapidly across many civil application domains. This paper aims to develop a farm monitoring system that incorporates UAV, LPWAN, and IoT technologies to transform the current farm management approach and aid farmers in obtaining actionable data from their farm operations. In this regard, an IoT-based water quality monitoring system was developed because water is an essential aspect in livestock development. Then, based on the Long-Range Wide-Area Network (LoRaWAN^®^) technology, a multi-channel LoRaWAN^®^ gateway was developed and integrated into a vertical takeoff and landing drone to convey collected data from the sensors to the cloud for further analysis. In addition, to develop LoRaWAN^®^-based aerial communication, a series of measurements and simulations were performed under different configurations and scenarios. Finally, to enhance the efficiency of aerial-based data collection, the UAV path planning was optimized. Measurement results showed that the maximum achievable LoRa coverage when operating on-air via the drone is about 10 km, and the Longley–Rice irregular terrain model provides the most suitable path loss model for the scenario of large-scale farms, and a multi-channel gateway with a spreading factor of 12 provides the most reliable communication link at a high drone speed (up to 95 km/h). Simulation results showed that the developed system can overcome the coverage limitation of LoRaWAN^®^ and it can establish a reliable communication link over large-scale wireless sensor networks. In addition, it was shown that by optimizing flight paths, aerial data collection could be performed in a much shorter time than industrial mission planning (up to four times in our case).

## 1. Introduction

The producers in the livestock industry have extensive areas of land and their assets have to be monitored constantly to ensure the operations are optimally maintained at all times. These lands are often situated in remote areas and many livestock and asset monitoring activities have to be performed manually on site by experienced personnel, which are traditionally time-consuming, costly, and oftentimes dangerous and lead to insufficient information about the condition of the farm and the health of the livestock.

In recent years, with the emergence of digital technologies such as wireless technologies, the Internet of Things (IoT), and low-power wide-area networks (LPWANs), significant advances have been made in farm management. Utilizing such technologies becomes even more beneficial and exciting when integrating them with unmanned aerial vehicles (UAVs). Such integration can be utilized to develop advanced livestock and farming monitoring systems.

Long Range (LoRa) is an LPWAN communication technology that enables a long-range transmission of data with low power consumption among things such as sensors [1]. Therefore, it can provide a reliable wireless communications link for remote areas with no or poor terrestrial wireless coverage [2] with affordable capital expenditure (CapEx) and operational expenditure (OpEx) [3]. In addition, it can connect sensors to the cloud and provide real-time communication for further data analysis and monitoring [4].

Generally, UAVs (also known as drones) can be categorized into two classes: high-altitude platform (HAP) and low-altitude platform (LAP). HAPs operate at an altitude above 17 km and stay for a long time at that altitude, like aircraft and balloons. On the other hand, LAPs operate at altitudes of tens of meters to a few kilometers and their endurance, size, and weight are less than HAPs, which can offer the advantage of high operating performance, reliability, and low CapEx [5], such as fixed-wing and rotary-wing drones. LAP drones are becoming increasingly used in a wide variety of cases, such as package delivery [6], surveillance [7], remote sensing [8], providing wireless communications [9], and precision agriculture [10].

From the wireless communications perspective, drones are capable of working as an aerial base station in the cellular network to provide a communication link for terrestrial users [11] or work as a relay in a wireless communication network [12]. However, in wireless sensor networks (WSNs), sensors have low transmission power and may not be able to wirelessly communicate over a long range. In such cases, applications of drones in the IoT have become very beneficial, where drones can operate as relays to enhance the connectivity and coverage in a WSN [13].

Despite promising opportunities to use drones in WSNs, some technical challenges need to be addressed, for instance, providing a reliable aerial communication link between sensor nodes and drones, network planning, sensor positioning, drones’ battery limitations, and trajectory optimization.

To minimize the need for manual operations, improve the quality of farm monitoring systems, and address some of the aforementioned issues, this study aims to develop a farm monitoring system (FMS) by integrating the IoT, LoRaWide Area Network (LoRaWAN^®^), and drone technologies. The FMS development is split into three main sub-modules, namely: (1) remote sensing development, (2) LoRaWAN^®^-based communication network development, and (3) UAV path planning optimization.

The first objective of this study is to develop a wireless-based water inspection system for monitoring five related parameters of water supply on the farm. The second objective is to integrate the LoRa gateway with a vertical take-off and landing (VTOL) drone for data collection from the distributed water quality sensor nodes and livestock collar tags and convey them to the cloud. The third objective is to optimize the drone flight path, based on the locations of sensors, to minimize its flight time and overcome the challenge of drones’ battery limitations. Hence, this study is a combination of hardware integration/development, measurement, and simulation. The utilized hardware was mainly based on off-the-shelf sensors and LoRaWAN^®^ modules, measurements were performed in real-world scenarios on a campus and in rural environments, and simulations were conducted based on assumptions of a large-scale livestock farm.

The key contributions of this study are summarized as follows:

- As water sources are an essential aspect in livestock development, a water inspection system was developed to gauge the quality of water using long-range wireless IoT technologies. The inspection sensors can collect data on a timely basis and transmit them to the LoRa gateway equipped onboard the drone.
- A multi-channel LoRa gateway, that is mounted on the drone, was developed to convey collected data by sensors to the cloud. In offline mode, when internet access is difficult to secure, the developed gateway works as local storage and stores the collected data and then pushes the data to the application server once an internet connection is available. For accurate network planning and implementation, the performance of the communications link was measured in different spreading factors (SFs), LoRa gateway modes, and drone speeds. Additionally, the Doppler effect was investigated at a higher flight speed than previous studies and it was found that LoRa has robust performance with a maximum drone speed of 95km/h and spreading factor of 12.
- All the key technologies, IoT sensors, and the LoRa gateway were successfully integrated into the developed VTOL drone to support farm monitoring operations. As a result, the developed system is a successful development in drone-based aerial data collection systems that provides a solution to tackle critical problems in large and rural farm management, aerial livestock monitoring, and collecting data from various IoT sensors.
- The drone flight path was optimized based on the traveling salesman problem (TSP) and enhanced particle swarm optimization (EPSO). In addition, the optimization results were compared with the most adopted drone flight operations in the real world. The results showed that path planning optimization is an effective solution to overcome drone battery limitations. Furthermore, the utilized method and algorithm can find the global optimum for the path planning problem which can significantly reduce the mission time of drones to collect data on large-scale farms.

The rest of the paper is organized as follows. A review of recent works is presented in Section 2. Section 3 describes the materials and methods used in developing the FMS which are divided into three subsections, remote sensing, LoRa-based communication network, and optimal path planning. Measurement and simulation results are presented and discussed in Section 4. Finally, the paper ends with a conclusion in Section 5.

## 2. Related Works

This section provides an overview of recent related works from the use of drones in smart farming to water inspection systems, LoRa aerial communications, and drone path planning optimization.

### 2.1. UAV Applications in Smart Farming

Drones are rapidly evolving in the field of agriculture and can perform numerous tasks, such as weed mapping [14], soil and crop status monitoring [15], pesticide spraying [16], diagnosis of insect pests [17], and artificial pollination [18].

In [19,20], an autonomous drone was developed for spraying pesticide and fertilizer on farms. Utilizing such a technique can enhance spraying efficiency and reduce pesticide usage and the risk of worker poisoning. The work in [21,22] used a drone for mapping. The maps can provide useful information such as in the monitoring of farm areas, soil conditions, and crop status. The authors in [23,24] developed methods for monitoring crop conditions by analyzing high-resolution crop data.

In addition, the utilization of drones has been addressed in the literature [25,26], [27,28] as an enabler for providing a reliable and cost-effective wireless communications solution for smart farming. By exploiting features such as autonomy, mobility, and adjustable altitude, drones can enhance a wireless network’s capacity, reliability, and energy efficiency [29].

The work in [30] investigated the feasibility of utilizing small commercial drones for indoor livestock monitoring. As a GPS signal is unavailable in indoor scenarios, the performance of visual simultaneous localization and mapping (VSLAM) algorithms was examined. The results showed that by equipping drones with the aerial VSLAM algorithm, indoor livestock monitoring can be feasible. The authors in [31] designed a system for sheep monitoring and tracking based on image processing techniques. The study demonstrated the hardware and software configuration for developing a drone and the measurement results showed that the system can reliably (with an accuracy of 89–97%) detect sheep on a farm. However, the drawback of the proposed system was the high power consumption of the utilized onboard companion computer to run the image processing algorithms.

For smart farming, the type of drone can be chosen based on factors such as the kind of environment, kind of application, required quality of service (QoS), flying altitude, movement speed, and maneuverability. For example, low-altitude drones are known as an appropriate and cost-effective approach for data collection from sensors in remote areas [32]. Compared to fixed-wing drones, rotary-wings drones weigh less, have more maneuverability, and can stay stationary over a given area. In contrast, fixed-wings drones fly faster, are able to carry more payload, and are more energy-efficient than the rotary-wing drones but need to move forward to remain airborne [5].

### 2.2. Water Quality Monitoring Solutions

Data collection can be considered as one of the main tasks of an IoT network. However, for large-scale WSN deployment, the task of data collection can be challenging and depends on the complexity of the geographical environment. Generally, the task of data collection can be divided into two types: static data collection and dynamic data collection [33]. In the static method, nodes of a WSN convey their collected data through a multi-hop network, while in the dynamic method, a movable data collector, like a drone, collects data of distributed nodes. Compared with the static methods, dynamic data collection can offer some benefits such as reducing the energy consumption of nodes for data transmission, enhancing network coverage, and extending the capability and flexibility of WSNs to operate in a different type of environment.

Mainly, there are two approaches for water quality monitoring in the farming industry, either via in situ measurement or by aerial imaging from the drone and, in rare cases, there is a combination of both. However, from the prior search, there is no reliable solution yet related to water quality monitoring, which relies on drone-based wireless communications.

The work in [34] used a drone-based thermal camera for estimation of water evaporation in a much finer spatial scale for irritation and water resource management. The work in [35] suggested using a multispectral image from the drone and compared the measurement with the in situ measurement by using portable water sensor equipment on site. The authors measured turbidity and chlorophyll a in their study. However, the authors concluded that they still have to rely on the in situ measurement and still need lots of aerial data to achieve reliable water quality measurement from a drone.

Similar work was carried out in [36], where the authors derived water quality parameters from the chlorophyll a, turbidity, and surface water temperature by using hyperspectral and thermal imaging. The data acquired were then compared against the in situ measurement using the WISP-3 ground sensor (to measure chlorophyll a and turbidity) and a laser thermometer to measure surface water temperature.

The earlier work to combine in situ and drone measurements can be traced from the project developed by Aerotestra [37]. The prototype works by immersing the water sensor while the drone is temporarily floating on a lake. The sensor measures the temperature reading, and there is a plan to extend the number of sensors, including pH, salinity, and dissolved oxygen.

Finally, the work in [38] attempted to use several water sensors mounted on the drone to measure the water quality parameters, such as DO, EC, pH, and temperature. This prototype has been shown to make semiautonomous in situ water quality measurements from predetermined waypoints. The results showed small measurement differences (maximum 3.8%) between the prototype and the in situ reading from a commercial probe.

### 2.3. Aerial IoT Communications

Under the IoT, several wireless technologies have been developed to cater for sensor-based communication, also popularly known as machine-to-machine (M2M) communications. The idea is to assign a dedicated network platform independent of the typical mobile/wireless broadband used to cater to human-based communications (such as streaming, data transfer, and voice communications). As a result, there are several variations of wireless IoT technologies and they are summarized in Table 1.

Recently, drones have been deployed as flying cellular base stations to provide reliable and energy-efficient IoT communications [39,40,41]. Studies show that such efficient utilization of drones can significantly improve the communications link between sensor nodes and drones, by enhancing the probability of line of sight communications [42] and reducing shadowing and blockage effects [43]. Moreover, the limited battery of the sensors will need considerably lower transmission power for transferring their data to the receiver sides [44]. However, most recent studies have focused on cellular-connected drones and less attention has been paid to studying the application of LPWAN technologies to drones.

On the other hand, the purpose of wireless propagation studies is to perform analysis in two crucial areas:

(1) Link budget: represents how much fade margin is available between a transmitter and a receiver to ensure a reliable wireless connection, and
(2) Coverage prediction: to estimate the maximum area covered based on the hardware configuration of a particular wireless technology [45]. Therefore, in the LoRa wireless deployment, a suitable wireless propagation model must be identified for the drone operator to ensure reliable connectivity and optimum wireless coverage.

Table 2 presents a summary of previous works on investigating LoRa performance versus the Doppler effect. From the table, it can be observed that the previous research works proved that LoRa is robust to Doppler shift, and five of these research works discovered that 80% of the packet is received under LoRa spreading factor 12 (SF12) via an experimental test on a moving car and human. Meanwhile, based on a simulation with a drone done by [46], drone speed at a maximum of 50 km/h does not affect the delay, jitter, packet loss, and output of LoRa. As the speed of VTOL drones is much faster than rotary-wing drones, there is a need to investigate the Doppler effects at a higher speed.

### 2.4. Drone Path Planning Optimization

One of the major limiting factors in designing a drone-based data collection system is its battery capacity (and consequently its flight time limitations). Based on the latest battery technology development, the utilization of lithium-ion batteries is considered the best option. Although the capacity of lithium-ion batteries is much larger than that of conventional batteries, the flight time of small drones is still limited to about 20–30 min [47]. Furthermore, increasing the battery size increases the overall weight of the drone. Hence, the problem of power source limitation is considered an unsolved problem for the use of drones in practice. A practical solution to overcome the challenge and enhance the efficiency of drone flight time is the optimization of drone path planning.

The authors of [48] studied the impact of drone speed on the performance of the data collection system. The study showed that the optimal drone speed depends on the distance between the UAV and sensor nodes, nodes’ transmission power, and amount of data. In addition, dynamic programming was used to optimize the drone speed and sensors’ transmission power, where the objective was to minimize the total flight time over a mission. Another factor that affects drone-based data collection performance is flight altitude. The authors of [49] optimized the drone’s flight altitude by minimizing the flight time and maximizing the number of successfully decoded bits in the uplink.

**Table 2:** Previous research works focusing on LoRa performance vs. Doppler shift.

Reference	Methodology	Result and Findings
[50]	Experimental: LoRa gateway located on 5th floor of a building LoRa node on a car roof Max speed (km/h): urban = 53, suburban = 57, rural = 70	SF7 is more vulnerable to the Doppler effect than SF12
[51]	Experimental: LoRa gateway on moving car roof LoRa node on a stationary car roof Max speed (km/h): line of sight = 120	Above 96% packet received at SF8 and SF12
[52]	Experimental: LoRa gateway mounted on a 24 m height tower LoRa node on a car roof LoRa node mounted on Lathe	LoRa node mounted on Lathe: at speed 11 m/s packet received is 36% at SF12 LoRa node on car roof: at speed 24 km/h packet data received is 28% at SF12
[53]	Experimental: LoRa gateway installed on a tripod with a height of 1.5 m LoRa node held by author LoRa node on a car roof	LoRa node held by the author: 80% packet received at 5 km/h speed LoRa node on a car roof: 85% packet received at 24 km/h
[54]	Experimental: LoRa gateway on top of 3-storey building LoRa node on a car roof	Above 85% packet received at speeds of 50 km/h, 60 km/h, 70 km/h and 80 km/h with SF12
[46]	Simulation: LoRa node on a drone State LoRa gateway Speed: 10, 25, and 50 km/h	The drone speed does not affect the quality of experience (QoE), which is the delay, jitter, packet loss, and output.

Drone path planning is considered a complicated optimization problem, in which the target is to find a superior flight route in an environment under different kinds of constraints. Over the past decade, a couple of methods have been proposed to solve the path planning problem for drones.

Generally, path planning methods can be classified into two groups: global (i.e., offline) and local (i.e., online) methods [55]. The global path planning methods are used to find a global optimal path in a known environment, and the generated path is considered as an expected trajectory to be followed by the drone during its mission. On the other hand, the local path planning methods are used for the cases in which the considered environment is fully unknown or partially known. In this case, drones should be equipped with onboard sensors and advanced control methods for real-time environment detection and path planning.

Recently, the works in [56,57] utilized deep reinforcement learning techniques, such as Q-learning, as a promising solution to solve the problem of real-time drone path planning in unknown dynamic environments. Alternatively, heuristics intelligent optimization algorithms have also been widely used in recent years to solve the local path planning optimization problems, such as graph-based algorithms [58], heuristic search algorithms [59], field-based algorithms [60], and intelligent optimization algorithms [61].

However, the computational complexity is one of the challenges in optimizing the drone path planning, which directly depends on the complexity of the environment profile and problem constraints such as kinematics and dynamics constraints. Fortunately, in large-scale remote farm scenarios, where the sensor nodes are arranged in advance and there are no high obstacles or a dynamic environment, the problem of UAV path planning can be solved based on global techniques, which consequently results in less complex problems which can be solved by metaheuristic intelligent optimization algorithms, such as the genetic algorithm (GA), the ant colony algorithm, and the PSO algorithm.

The authors in [60] focused on global path planning under a static environment and used the GA to optimize the drone trajectory under maneuverability constraints. The work in [62] used the wolf pack algorithm to find an optimal path for rotor-wing drones by considering multi-objective cost functions in real and fake 3D spaces. To improve the performance of the wolf pack algorithm, the crossover and mutation operators of the GA were applied to the algorithm.

To further reduce the complexity of selecting the optimal path, the path planning problem can be converted to the TSP [63]. The optimization methods for solving the TSP include metaheuristic algorithms and fuzzy neural networks. The authors in [64] modeled the path planning problem for drone-aided data collection as the TSP and proposed a fast path planning with rules algorithm based on grid division to minimize the path distance, where the paths in the divisions are combined based on the line precedence principle.

Despite the popularity and wide usage of intelligent optimization for path planning problems, the algorithms should overcome some destructive phenomena such as local optimum trapping and early convergence. In this regard, PSO is an optimal algorithm to solve the TSP due to its powerful ability for local and global search (i.e., exploration and exploitation), which can be considered an efficient algorithm for drone path planning in a large-scale WSN.

## 3. Farming Monitoring System Concepts and Methods

**Figure 1:**
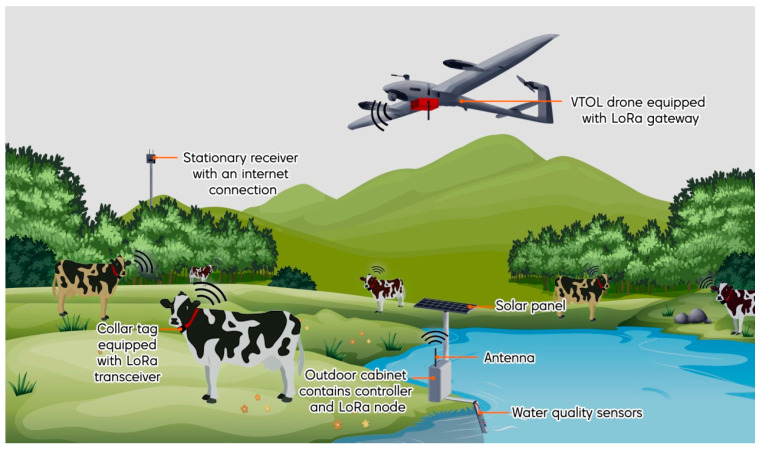
Conceptual design of the developed farm monitoring system.

The main purpose of the designed system is to enable drones to fly optimally over large farms and collect data from different sensors deployed on the farm and convey them to the cloud. Figure 1 depicts the conceptual design of the FMS. The FMS development consists of four main phases, development of the water inspection system, LoRaWAN^®^-based network planning, integrating the LoRaWAN^®^ gateway into the drone, and drone path planning optimization. The following subsections describe the methods used and works conducted to achieve the objectives of this study in detail.

### 3.1. Sensor Development and Deployment

In this study, two types of sensors were deployed on the farm: (i) water inspection sensors to monitor the quality of water supply on the farm, and (ii) collar tags to monitor the behavior and health status of livestock. The development of the collar tag is out of the scope of this paper. However, for practical implementation, several collar tags are available on the market. One of them is the SODAQ solar powered LoRa cattle tracker V2, which is manufactured by Sodaq^®^ in Hilversum, Netherlands [65]. The collar tag is equipped with a LoRa module microchipRN2903, uBlox 8M GPS, and LSM303AGR accelerometer. Hence, in the simulations, a transmission range of 500 m was assumed for collar tags, which is compatible with the transmission range of water sensor nodes.

**Figure 2:**
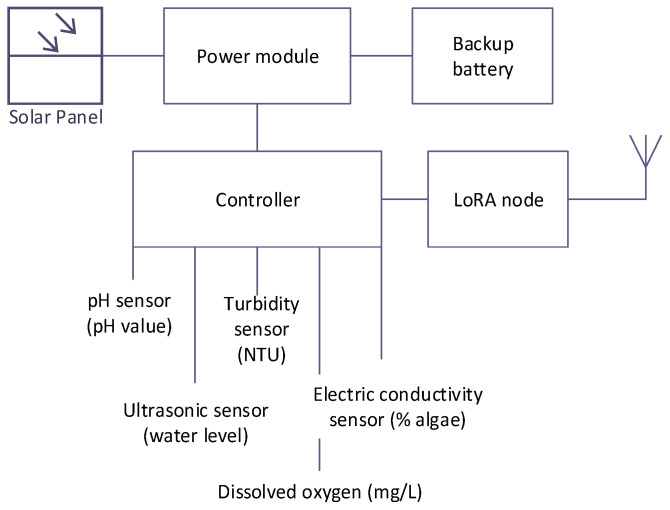
Overview of sensors and hardware for the water quality monitoring system.

The development of the water inspection system took place in multiple stages. Firstly, the conceptual design of the system and the required hardware were determined. This was then followed by the development of individual components to produce the water inspection system. Finally, the water sensors, wireless communication module, controller, solar panel, and power module were integrated, as shown in Figure 2.

**Figure 3:**
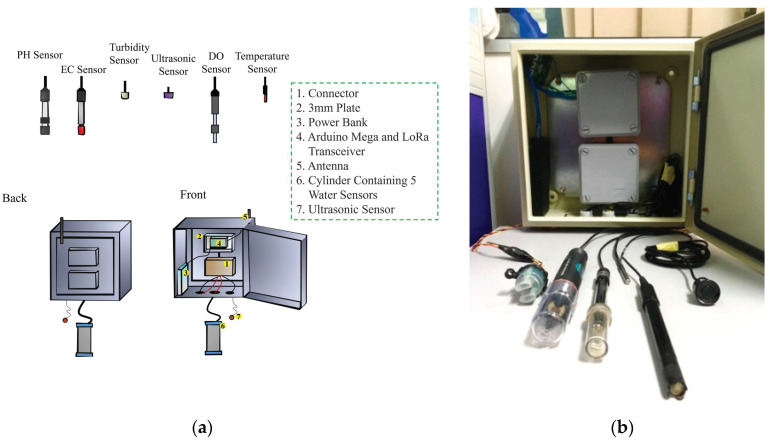
(**a**) Water node cabinet technical layout and (**b**) the integrated water quality monitoring system.

The utilized water sensors can sense five water quality parameters, including an ultrasonic sensor (to measure water level), pH sensor (to measure acidity/alkalinity), dissolved oxygen sensor, turbidity sensor (to measure water clarity), and electrical conductivity (EC) sensor (to measure the presence of algae). Figure 3 depicts the layout and the developed water quality monitoring system.

The controller acts as the center to manage the water quality data. This includes handling the data from sensors and passing them to the target device, which in this case is the LoRa transmitter. The controller captures all the data from the sensors and converts them into specific units to be shown in the interface. The controller also integrates the LoRa node because it is equipped with the LoRa chip from Semtech. When all the data from the sensors involved have been captured, the data are then packed according to the LoRa format to be sent to the LoRa gateway. The frequency of sending data to the gateway can be set from the controller itself.

The distribution of sensors in the field was based on the following factors: (i) location of farm water tributaries from the river, (ii) location of livestock watering pools, and (iii) distribution of herds on the farm.

**Figure 4:**
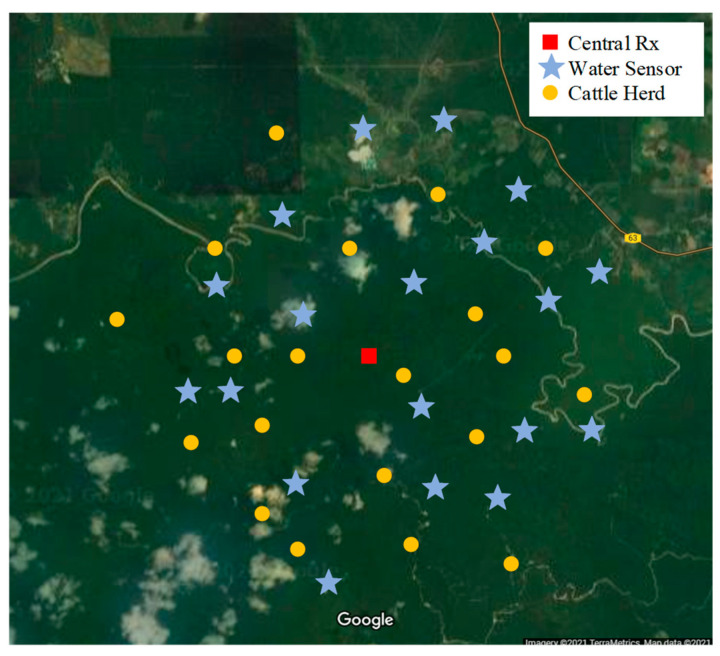
A schematic of the position of the sensors on the considered farm.

Figure 4 depicts a schematic of sensor deployment in the considered farm. The farm is a livestock complex with a capacity of 1000 dairy and beef cattle located in Pahang state, Malaysia. The farm area is about 3500 acres with an approximate radius of 7 km, which is considered a large-scale livestock farm [66].

The point marked with the square indicates the location of the central receiver, which is also the takeoff and landing platform of the drone. The points marked with asterisks indicate the location of the water inspection sensors (seven sensing units located in the river and 12 sensing units located in the livestock watering pools), and the points marked with circles show the location of cattle herds (20 points), with each herd containing an average of 42 cows [67].

To estimate the position of the herds, the movement of the cattle was monitored for several consecutive days.The location information of cattle movement was extracted from the collar tags. The data analysis showed a probability distribution of cattle movements, which after fitting to a normal distribution, the mean value was extracted and used as estimated position of herds.

### 3.2. LoRaWAN^®^-Based Communication Network

As described in the previous subsection, the proposed system was developed based on LoRaWAN^®^ wireless communication technology. The system consists of two main device types: the sensing unit, known as the end node (EN), and the central unit, known as the gateway (GW). Both units were developed based on either Semtech SX1272 or SX1301 LoRa chipsets, enabling the development of class A LoRaWAN devices. The EN is responsible for data collection and pre-processing. These pre-processed collected data are then be transmitted from these ENs wirelessly using the LoRa signal to the GW. The GW then forwards the collected data through an internet connection to the application server to be further processed and visualized by the end user. It should be noted that both the GW and ENs operated at the 915 MHz band. For simplicity and comparison purposes, these devices were configured at either LoRa mode 1, utilizing a 125 kHz bandwidth with an SF of 12, or LoRa mode 10, utilizing a 500 kHz bandwidth with SF7.

Two types of GW were utilized throughout the development process, namely a single-channel GW and a multi-channel GW, to specify the most suitable type for the deployment area. These two types GWs mainly differ in terms of cost, packet transmission reliability, and the maximum supported ENs. For instance, the single-channel GW is known for its low cost, support of few ENs (maximum of 4 ENs in our case), and the capability of handling one LoRa mode at a time for all ENs in the network. However, the latter represents a disadvantage that would eventually result in reduced performance and high packet loss. In contrast, the multi-channel GW can simultaneously operate at multiple frequency channels (up to eight), supporting a larger network of connected ENs and an improved packet transmission reliability. In addition, the multi-channel GW supports ENs operating at different spreading factors by automatically adapting the network, enabling what is known as the adaptive data rate (ADR) mode. In this regard, during the measurements, we evaluated and compared the performances of both GWs in terms of packet delivery rate (PDR) under different SFs and transmission time intervals.

**Figure 5:**
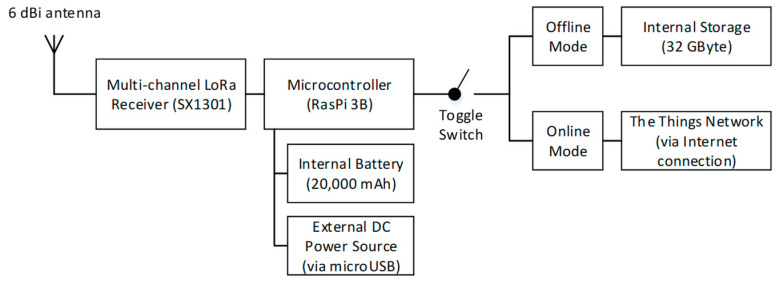
Block diagram of the modified LoRaWAN^®^ multi-channel GW.

**Figure 6:**
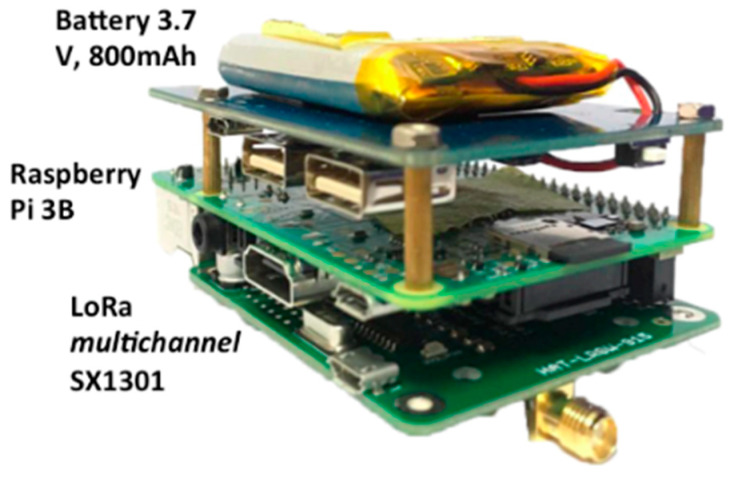
Components of the LoRaWAN^®^ multi-channel GW.

One of the drawbacks of the utilized multi-channel GW is that it does not support operating in areas where internet access is difficult to secure, i.e., it does not support operating in offline mode. Accordingly, it was further developed to enable offline data collection mode. This was achieved by using the same chipset of the multi-channel with a different hat design based on a Raspberry Pi and a second server was installed in the gateway to save the LoRa packet from the node locally. Thereby, it acts as a local storage device and a mini server that stores and pre-processes the data while in offline mode. The GW then pushes the data to the application server once an internet connection is available, in which the operation can be made by using a toggle switch on the gateway. Figure 5 and Figure 6, respectively, show the block diagram and the components of the modified LoRaWAN^®^ multi-channel GW, and Table 3 lists the technical specifications of the LoRa multi-channel GW.

To identify the capability of the LoRa wireless communication and coverage prediction, a CloudRF^®^ simulator was used. CloudRF^®^ is an online radio planning tool that can perform link budget and coverage prediction by considering the surrounding effect. CloudRF^®^ also offers configurable options to simulate the conditions and characteristics of real equipment by employing a high-resolution topographic graph. Further descriptions of simulation parameters and measurement methods are provided in related parts of Section 4.

### 3.3. Drone Path Planning Optimization

In this study, an industrial agricultural hybrid fixed-wing VTOL drone, known as AeroHawk [36], was utilized to perform measurements. The selected VTOL drone has the advantages of both fixed-wing and multi-rotor drones, which can take off and land vertically without a long runway while having the leverage of more extended range and endurance due to the aerodynamic efficiency generated from the wings during flight. This key feature is important for farmers with a large area of land.

To find the shortest flight path for the drone, first, the path planning problem was modeled as the TSP, and then both PSO and EPSO algorithms were used to solve the problem.

In the TSP, the nodes and distance between the nodes are given, and the object is to find the minimum overall distance to travel from one node to other nodes and back to the starting node without crossing a node twice. According to graph theory, the TSP solves the Euclidean metric to find a Hamilton loop with the smallest weight in the weighted completely undirected graph. However, the TSP belongs to the class of non-deterministic polynomial (NP) problems. To reduce the computational complexity, metaheuristic algorithms such as the GA, PSO, ant colony optimization (ACO), and neural network (NN) have been used. In this study, we used PSO because of its advantages such as strong robustness, simulation evolution, ability to store past iterations, and easy implementation.

The PSO algorithm works in such a way that, first, all particles are scattered randomly on the search space and every particle calculates the objective function based on its position in the search space. Then, each particle computes its next movement direction based on a combination of information about its current position, its best position that it has experienced so far, its current velocity, and information from one or more of the best particles in the swarm. Then, particles move, one step of the algorithm ends, and, in case of necessity, the above steps are iterated until the algorithm finds the optimal solution. In our simulation, we set the PSO algorithm to stop its execution when meeting the defined number of function evaluation (NFE) value, which is defined as

(1) $$NFE\left(t\right)=n_{pop}+n_{pop}\times t=n_{pop}\left(1+t\right),$$

where $n_{pop}$ is the population size (swarm size), and t is the number of iterations.

To formulate the behavior of particles, assume there are n particles in the swarm, where the position and velocity of ith particle at time t are denoted as $x^{i}$ and $v^{i}$, respectively, for i∈{1, 2, …, n}. $x^{i,\;best}\left[t\right]$ is the best position that the ith particle has experienced so far and $x^{gbest}\left[t\right]$ is the best position in the swarm’s experience. In every iteration (generation), the swarm updates its best position (based on objective value) which is known as the global best, and each particle updates its best solution (i.e., personal best) and computes its next position as follows:

(2) $$v^{i}\left[t+1\right]=wv^{i}\left[t\right]+c_{1}r_{1}\left(x^{i,\;best}\left[t\right]-x^{i}\left[t\right]\right)+c_{2}r_{2}\left(x^{gbest}\left[t\right]-x^{i}\left[t\right]\right),$$

(3) $$x^{i}\left[t+1\right]=x^{i}\left[t\right]+v^{i}\left[t+1\right],$$

where w is the inertia coefficient, $c_{1}$ and $c_{2}$ are cognitive and social acceleration coefficients, respectively, and $r_{1}$ and $r_{2}$ are random numbers in the range of 0–1.

**Figure 7:**
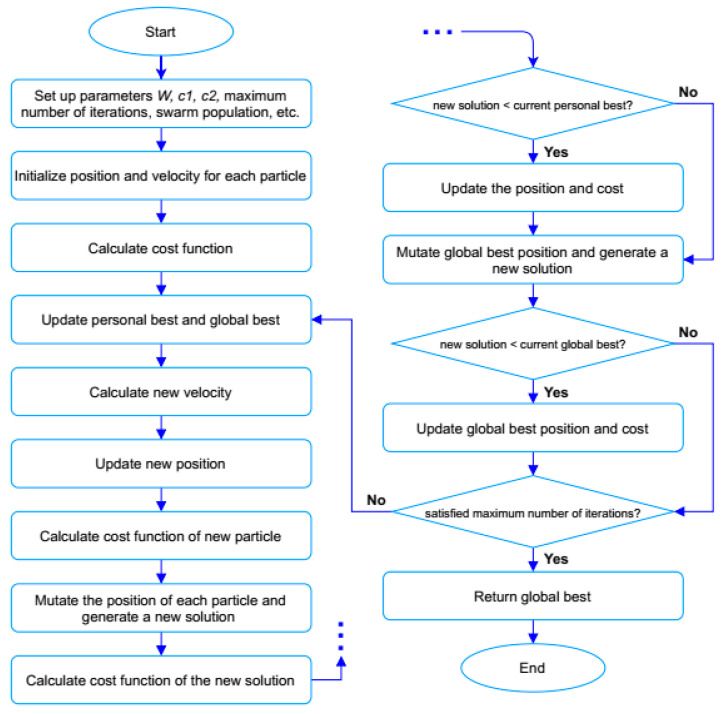
Flowchart of enhanced particle swarm optimization algorithm.

The weakness of the classic PSO algorithm in solving the TSP is that it falls into the trap of local optimums. To address this problem, we applied three mutation operators, called swap, reversion, and insertion, into the PSO algorithm, and the modified PSO algorithm is called EPSO. Figure 7 shows the flowchart of the EPSO algorithm which was implemented in MATLAB. The initial parameters are generated through the swarm initialization. As the adjustment of w, $c_{1}$, and $c_{2}$ plays an important role in the performance of the PSO algorithm (e.g., it directly affects the convergence speed of the algorithm to the best cost function) the w, $c_{1}$, and $c_{2}$ parameters were selected as [68]

(4) w=χ,

(5) $$c_{1}=\chi \phi_{1},$$

(6) $$c_{2}=\chi \phi_{2},$$

where variables $\phi_{1}$ and $\;\phi_{2}$ are positive numbers, $\phi \equiv \phi_{1}+\phi_{1}>4$, and

(7) $$\chi =\frac{2}{\phi -2+\sqrt{\phi^{2}-4\phi }}.$$

According to [68], the optimal values for the above parameters are $\phi_{1}=\phi_{1}=2.05$, w=0.7298, and $c_{1}=c_{2}=1.4962$.

**Figure 8:**
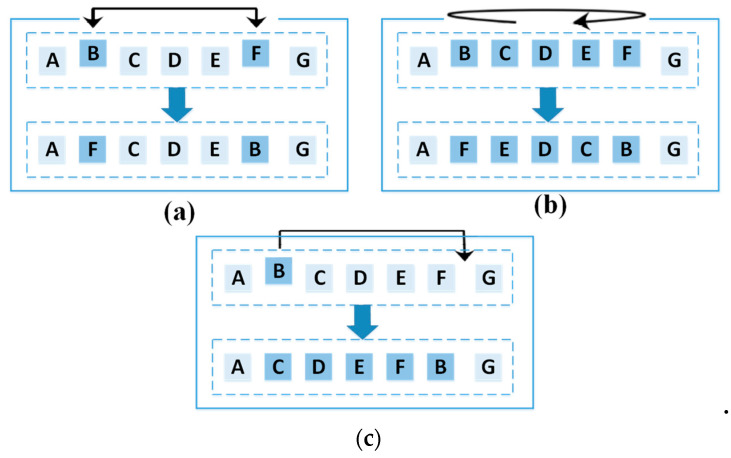
A schematic of the utilized mutation operators, (**a**) swap, (**b**) reversion, and (**c**) insertion.

**Figure 9:**
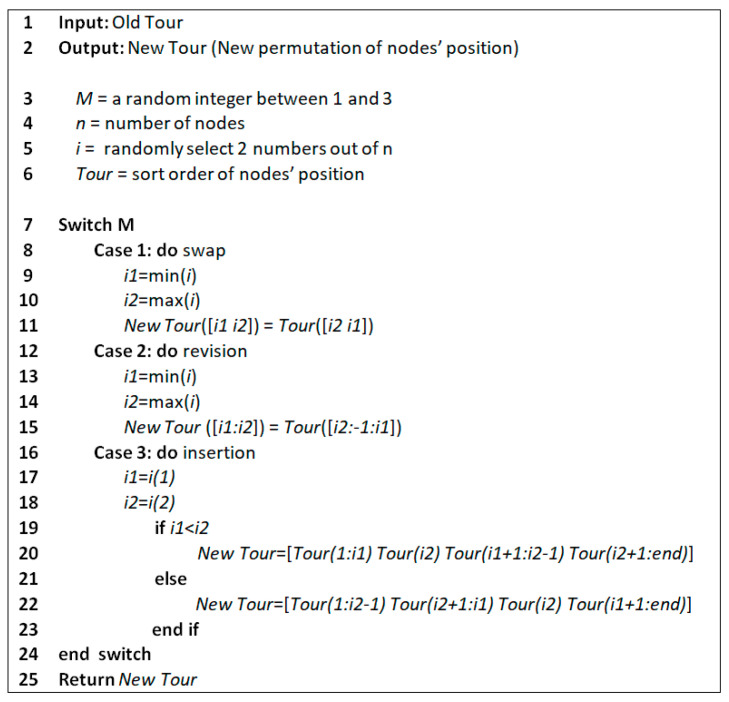
Pseudocode of the utilized mutation operators (swap, reversion, and insertion).

Figure 8 depicts how the utilized mutation operators operate. The swap operator exchanges the places of two randomly chosen nodes. The reversion operator exchanges the places of all nodes in a randomly selected range. The insertion operator moves a randomly chosen node to another random place. Figure 9 presents the pseudocode of the utilized mutation operators.

## 4. Results and Discussions

### 4.1. Coverage Analysis and Path Loss Limits of LoRaWAN^®^ Wireless IoT Communication

**Figure 10:**
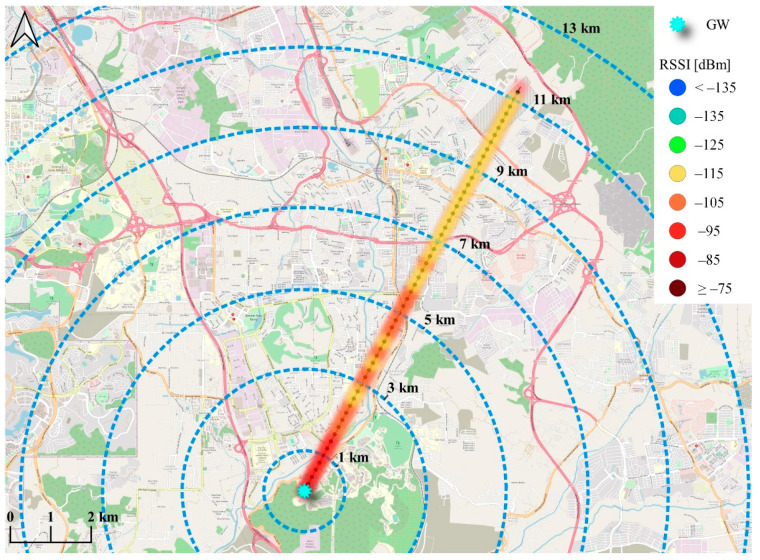
Measurement route at the UKM campus area (Bangi, Malaysia) and the heatmap of LoRaWAN^®^ coverage throughout the measurement route.

The purpose of this section is to identify the capability of LoRaWAN^®^ technology to provide aerial wireless coverage, analyze the maximum achievable coverage when operating on-air via a drone, and identify the most suitable path loss (PL) model for the study area. Accordingly, a set of received signal strength indication (RSSI) measurements were taken at the National University of Malaysia (UKM), Bangi campus. The measurement setup consists of two devices; one GW mounted on a tripod at 2 m above ground level (AGL), near the Faculty of Engineering and Built Environment (FKAB), and an EN mounted on a drone. During the measurements, the drone took off vertically and maintained a height of 200 m AGL. The drone then moved horizontally through the measurement path shown in Figure 10 and transmitted the data packets every 200 m. The average RSSI value for the received packets at each of the 56 points was then recorded at the GW side. The GW and EN operated at the 915 MHz band and used SF12 at 125 kHz BW with a fixed transmission power of 20 dBm. Meanwhile, the antenna was an omnidirectional antenna of 2 dBi and 0 dBi gain for the GW and EN, respectively.

As shown in Figure 10, the communication distance achieved was more than 10 km using the default setting. It was also noted that the signal strength was significantly below the LoRa receiver sensitivity of −137 dBm. The worst recorded signal was −110 dBm, which translates into coupling loss of 130 dB, calculated as $P_{\mathrm{Tx}}-\min\left(\mathrm{measured}\;\mathrm{RSSI}\right)$. The latter is below the theoretical maximum coupling loss (MCL) of 157 dB [69] of LoRa-based systems. As such, higher coverage is expected using the utilized configurations. Hence, it can be observed that the LoRaWAN^®^-based aerial wireless communication system represents a suitable and reliable communication option for the intended application.

To further illustrate losses in the propagated signal due to factors such as shadowing, PL was calculated and compared with other reference empirical PL models. These models included a free space PL (FSPL) model as a baseline and two popular PL models based on Cloud-RF^®^. The latter models include the Longley–Rice irregular terrain model (ITM) and ECC-33. PL was first calculated from the measured received power ($P_{Rx}$), equivalent to the measured RSSI in dBm, using Equation (8) [70]:

(8) $$PL\left(dB\right)=P_{Tx}-P_{Rx}+G_{Tx}+G_{Rx}-L,$$

where, $P_{Tx}$, $G_{Tx}$, $G_{Rx}$, and L are the transmitted power in dBm, transmitter antenna gain, receiver antenna gain, and other losses on both the transmitter and receiver’s side, respectively. L is neglected in the calculation and assumed to be zero. FSPL was then calculated using Equation (9) [70]:

(9) $$PL_{FSPL}=20log_{10}\left(f\right)+20log_{10}\left(d\right)+32.44,$$

where f is the frequency in MHz and d is the separation distance between the transmitter and the receiver in km.

**Figure 11:**
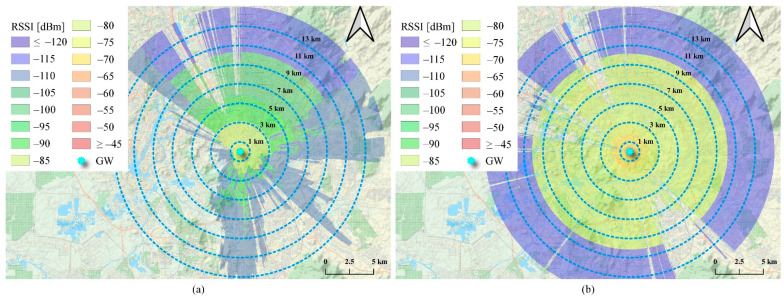
Heatmap of LoRaWAN^®^ coverage at UKM campus based on Cloud-RF^®^ PL simulations. (**a**) LoRaWAN^®^ coverage based on the ITM PL model. (**b**) LoRaWAN^®^ coverage based on the ECC-33 PL model.

Finally, for Cloud-RF^®^, simulations were performed using the same measurement parameters used during the measurements performed at the campus (see Figure 11). The tool works by assigning the area type-dependent parameters based on the area maps, considering the type of signal path area. It also considers the knife-edge diffraction impact and the irregular terrain impact on the transmitted signal (for the ITM) in the calculation process for more accurate PL predictions. Once simulations were complete, the PL data were extracted for each measurement point using the best server analysis feature of Cloud-RF^®^.

**Figure 12:**
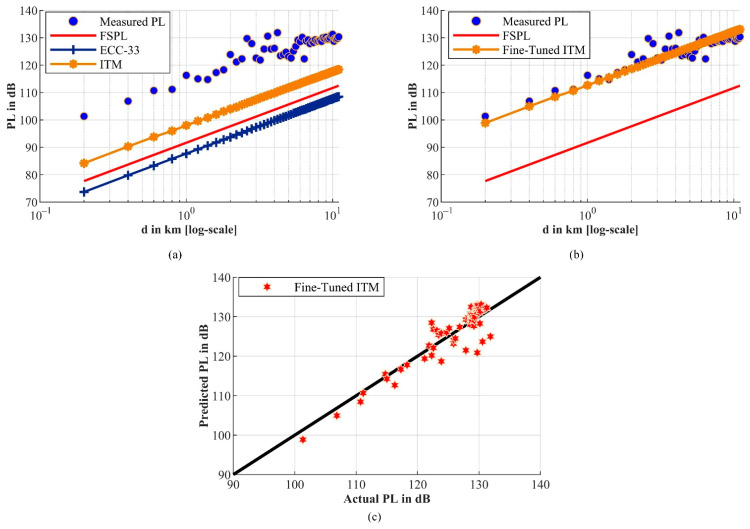
(**a**) Measured PL vs. predicted PL from FSPL (baseline) and two Cloud-RF^®^-based PL models. (**b**) Measured PL vs. predicted PL from FSPL (baseline) and fine-tuned ITM PL model. (**c**) Actual vs. predicted PL correlation scatter plot for the fine-tuned Cloud-RF^®^-based ITM PL model.

Figure 12a shows the actual measured PL compared against the extracted PL measurements for the two evaluated PL models based on the Cloud-RF^®^ tool simulations in addition to the calculated FSPL as a baseline. From the results, it is evident that the data do not fit FSPL. On the other hand, it can be seen that Cloud-RF^®^-based PL models tend to underestimate the PL predictions. Therefore, a set of common prediction error evaluation metrics were also considered to evaluate these modes further, as shown in Table 4.

These metrics include mean squared error (MSE), root mean squared error (RMSE), mean absolute error (MAE), mean arctangent absolute percentile error (MAAPE), coefficient of correlation (R), and squared R (R2), also known as the coefficient of determination. Based on these evaluation metrics, it can be emphasized that all models show poor prediction performance, with ECC-33 showing the worst prediction performance. As such, the ITM was selected and was then fine-tuned to minimize the prediction error. In this regard, the RMSE was considered the objective function to be minimized by adding a correction factor (S) to the ITM PL response in dB. The fine-tuning step led to S = 14.664 dB, which significantly improved the ITM’s prediction performance, as shown in Figure 12b,c and Table 4. For example, in terms of MSE, a 96.2% improvement was observed for the fine-tuned ITM.

Similarly, the performance was also improved in terms of other metrics, except for R, showing a good correlation, as shown in Figure 12c. Hence, it can be concluded that the Cloud-RF^®^-based ITM (after fine-tuning) might represent the most suitable model for LoRaWAN^®^-based coverage prediction and planning in the considered study area. However, we suggest that further enhancements or new PL model proposals are required to provide highly accurate predictions. Based on the observations in this section, we utilized the fine-tuned ITM PL model for coverage planning in the considered rural farm.

### 4.2. LoRaWAN^®^ Performance under Single-Channel and Multi-Channel GW

As discussed earlier, the performance of single-channel and multi-channel GWs differs in some aspects, such as packet transmission reliability and the maximum supported ENs. In this section, these differences in performance are further investigated to understand PDR variation by comparing both GWs, under different SFs (7 and 12). In addition, the comparison also considers the time interval of the LoRa transmitter (i.e., the duty cycle period) and the number of ENs operating simultaneously (to test how these GWs cope with various node sizes).

**Figure 13:**
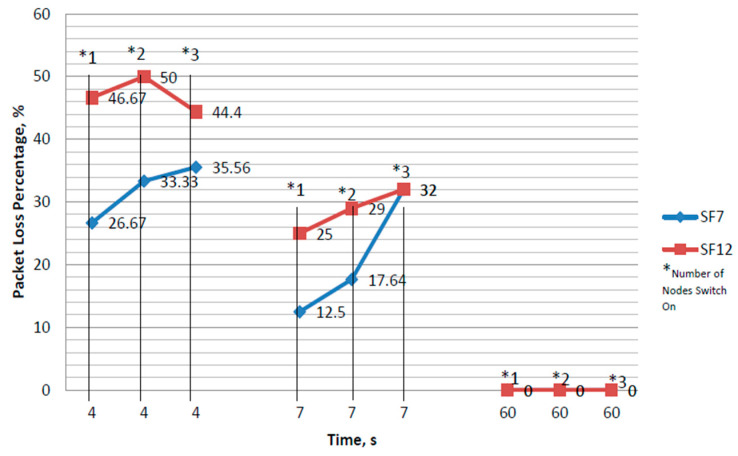
The percentage of packet loss during data transmission between the LoRaWAN^®^ GW and EN.

**Figure 14:**
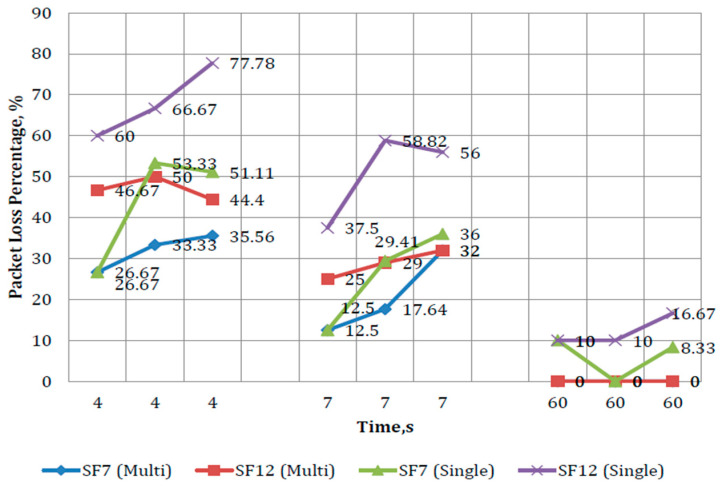
Performance comparison between the LoRaWAN^®^ multi-channel and single-channel GWs.

From the results shown in Figure 13 and Figure 14, it can be observed that the SF, duration of sleep time, and the number of ENs have a significant impact on the PDR. Furthermore, the multi-channel GW performed better than the single-channel GW due to its capability to support many ENs (up to 800). On the other hand, by increasing the SF, number of operating ENs, and decreasing the sleep time, the packet loss increased.

### 4.3. LoRaWAN^®^ Performance under Different Drone Speeds

**Figure 15:**
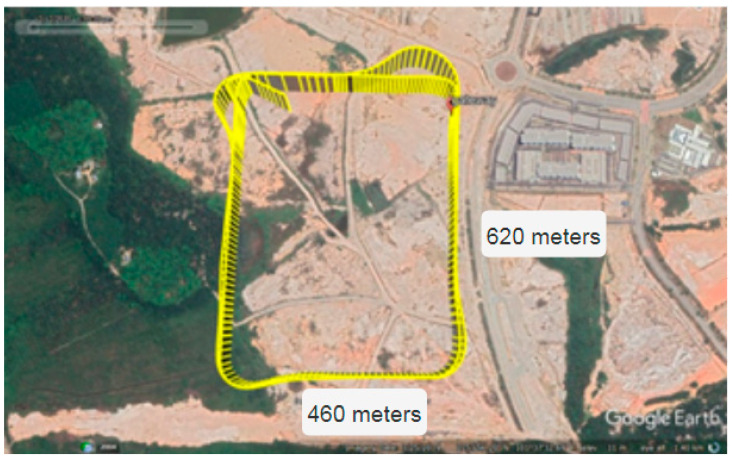
Aerial view of the location (Cyberjaya, Malaysia) for drone speed measurement.

To quantify the Doppler effect on the LoRaWAN^®^-based aerial communication, the performance of the system was examined under different drone speeds. To increase the safety and maneuverability of the drone, measurements were performed in an open and uninhabited area, as shown in Figure 15. The multi-channel GW was stationary, located on the ground, while the EN was mounted on the drone to perform a real-time data update. The data included several measurements, particularly on the percentage of the packet received for this experiment.

Data from the EN were transmitted every 5 s, while data were received by the GW every 9 and 7 s for the case of SF12 and SF7, respectively. The difference in time required to receive data in SF12 and SF7 is due to the on-air time, as SF12 on-air time is longer than SF7. In addition, the drone flew at different speeds with a ±5 km/h variation due to unpredictable wind direction during measurement and a fixed altitude of 100 m above the ground.

**Figure 16:**
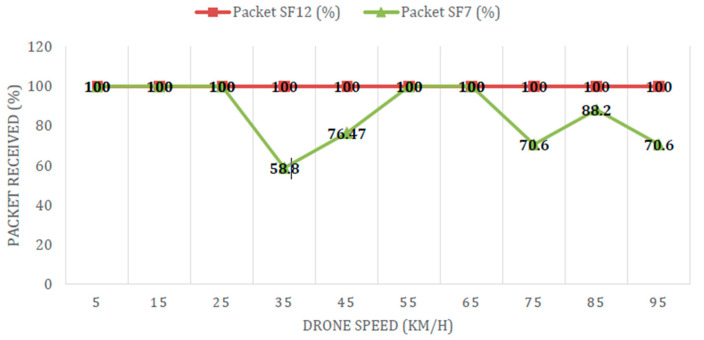
Performance of LoRa multi-channel gateway against different drone speeds.

Figure 16 shows the percentages of packet delivery at different speeds for SF12 and SF7, which are 100% and 77.27% (on average), respectively. Results show the robustness of SF12 towards the Doppler effect, while SF7, on the other hand, is proven to be sensitive to the drone speed, especially at a speed of 35 km/h and above. This measurement offered a new insight into the performance of LoRaWAN^®^-based aerial communication because the previous study in [46] was limited to 50 km/h.

### 4.4. LoRaWAN^®^ Coverage Analysis for Optimal GW and EN Height Planning in a Rural Farm Area

**Figure 17:**
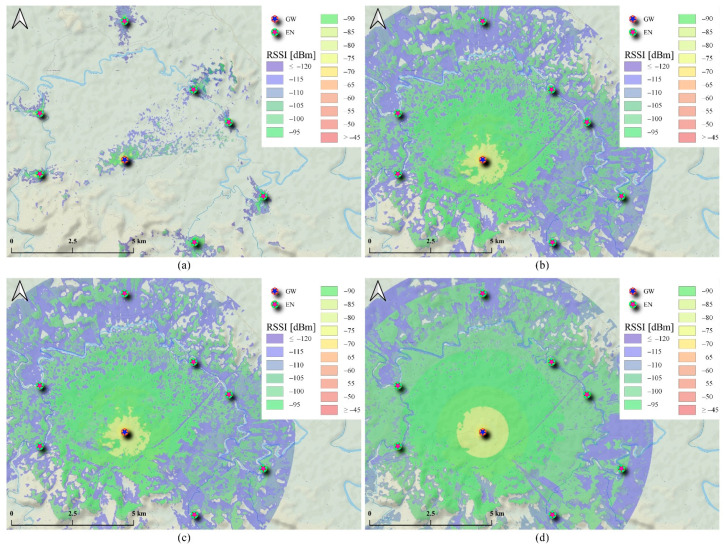
LoRaWAN^®^ coverage prediction in a rural farm area (Pahang, Malaysia) using the fine-tuned ITM PL model in Cloud-RF^®^ based on different GW and EN height configurations. (**a**) GW in idle state (on the ground) and EN at 1 m AGL. (**b**) GW at 50 m AGL and EN at 1 m AGL. (**c**) GW at 100 m AGL and EN at 1 m AGL. (**d**) GW at 150 m AGL and EN at 1m AGL.

One of the challenging tasks for the LoRaWAN^®^ GW mounted on a drone during operation is identifying optimal configurations, specifically for Tx/Rx height, to guarantee reliable communications between the GW and ENs in the network. As such, this section analyzes the coverage of the deployment area based on Cloud-RF^®^ simulations at different GW and EN height configurations, as shown in Figure 17. The simulations were based on the fine-tuned ITM PL model and used the configurations specified in Table 5. To simplify the simulation and the discussion, only seven ENs (water quality sensors located in the river) have been considered at this stage and the results can be easily generalized to all other ENs.

Three different GW heights were considered in addition to the idle state, where the GW was placed on the ground, to justify the need for such system requirements. Additionally, the coverage of each of the seven sensing nodes was simulated separately at minimal heights to justify the need for the drone-based system further (Figure 17a). Based on the coverage results shown in Figure 17, it can be observed that GW height has a significant impact on overall achievable coverage. Therefore, this also justifies the need for a drone-based system to guarantee reliable communications.

**Figure 18:**
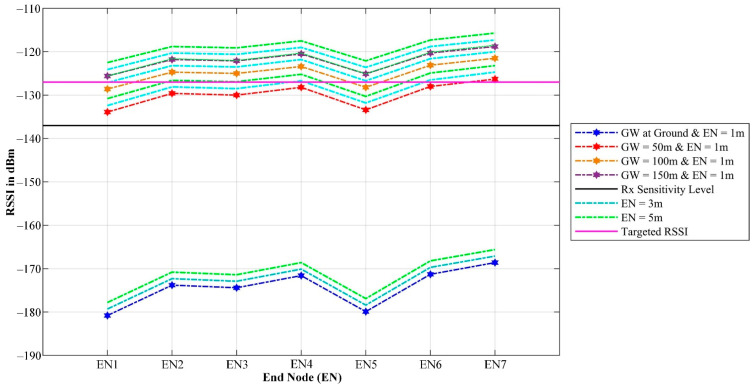
Predicted RSSI using the fine-tuned ITM PL model in Cloud-RF^®^ with various GW and EN height configurations.

To further illustrate the impact of GW and EN height, the predicted RSSI at each of the seven ENs was extracted from the simulations and plotted as shown in Figure 18. Based on the plot, it can be seen that switching the GW height to 50 m would result in an RSSI that is slightly above the LoRaWAN^®^ receiver sensitivity level. Meanwhile, increasing the GW height to 100 m or 150 m would result in an average improvement from 3 dB to 7 dB. On the other hand, changing the EN height showed an additional minor improvement.

Finally, to compensate for other signal-degrading factors that are not considered in the Cloud-RF^®^ simulations, such as temporal fading and dense foliage impact, we can assume that the optimal RSSI required for the system should be kept 10 dB above the minimum sensitivity level. As such, it can be concluded that the optimal GW and EN heights are 150 m AGL and 1 m AGL, respectively, for the final deployment area.

### 4.5. Path Optimization

**Figure 19:**
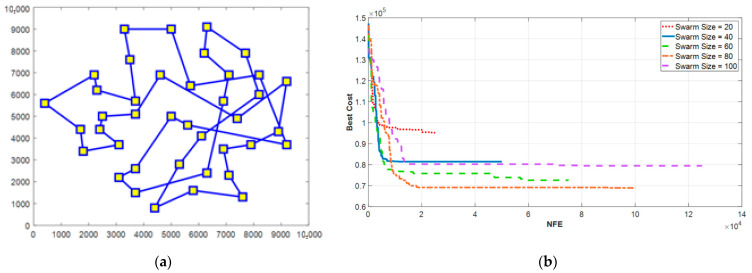
(**a**) The optimal flight path calculated by the PSO algorithm and (**b**) the performance of the PSO algorithm under different sizes of the swarm.

To find the minimum traveling path for the aerial data collection on the considered farm, the positions of the deployed sensors were used as the input of the TSP and, in addition, the PSO and EPSO algorithms were utilized to solve the problem. Figure 19a shows the result of the PSO algorithm where a swarm size of 100 was considered during the simulation. As seen from the result, the paths intersect and the algorithm cannot find the global optimum. The weakness of the original PSO algorithm in solving the TSP is that the algorithm soon falls into the trap of local optimum. To further evaluate the performance of the algorithm, several swarm sizes were examined (i.e., 20, 40, 60, 80, and 100), and the maximum number of iterations was set to 1250.

Figure 19b shows that even by changing the swarm size, the algorithm cannot converge to the best cost function and even when increasing the swarm size to 100, the cost function increases and becomes worse. The main reason for this behavior is that the initial answers of evolutionary algorithms are reached randomly and, because of the complexity of the TSP, especially when the number of nodes increases, the particles mainly exploit their local optimum neighborhood, instead of exploring the entire search space and finding the global optimum.

**Figure 20:**
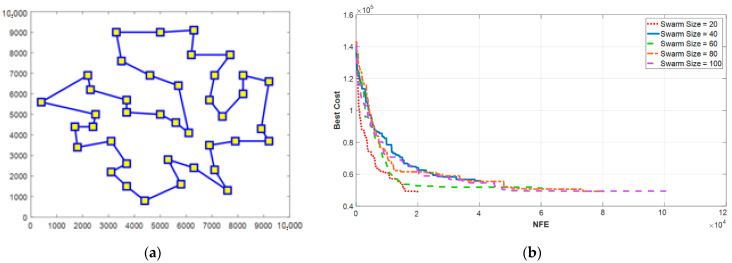
(**a**) The optimal flight path calculated by the enhanced PSO algorithm and (**b**) the performance of the EPSO algorithm under different sizes of the swarm.

To solve the challenge, the idea of mutation from the GA was used and applied to the TSP. Therefore, in each iteration, the EPSO algorithm generates some random new solutions for both personal and global cases, resulting in a higher exploration rate. Figure 20a shows the optimal flight path without any intersection, and Figure 20b presents the performance of the ESPO algorithm when the size of the swarm changes. As can be seen from the results, regardless of the swarm size, the algorithm converges to the best minimum cost function. By comparing the results of Figure 19b and Figure 20b, EPSO can reduce the cost function by 35% compared to PSO. In other words, the EPSO algorithm was able to reduce the total length of the route from 79 km to 49 km.

**Figure 21:**
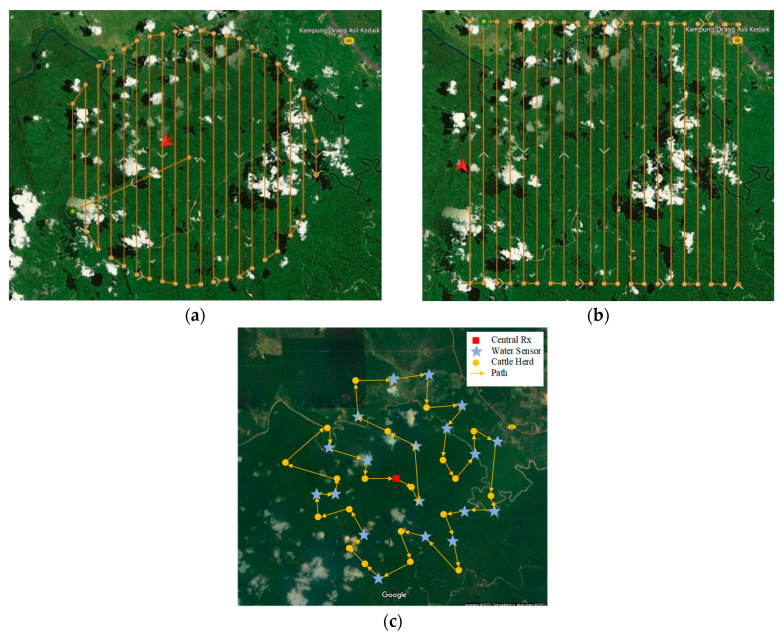
(**a**) Circular model path planning, (**b**) square model path planning, and (**c**) optimized path planning.

Industrial farming drones usually follow a series of predefined routes in their flight mission planner software to sweep the entire farm, e.g., zigzag, square, and circular routing patterns. To justify the need for path planning optimization in large-scale drone-based data collection, the Ardupilot^®^ mission planner was used and the flight path was planned in square and circular modes. Figure 21a,b shows circular and square path planning, respectively, and Figure 21c depicts the optimized drone path for the considered farm. Furthermore, to ensure that the drone can reliably collect data from all the ENs, the distance between adjacent routes was set to 500 m. According to the results, the circular and square paths’ total length was about 175 km and 212 km, respectively.

Table 6 compares the performance of the developed system under different flight mission modes. In the calculation, a maximum flight time of 60 min is assumed, following the specifications of the utilized drone and the small industrial VTOL drones [71]. According to the results, by optimizing the flight path, the same mission can be performed in a much shorter distance (49 km) and a shorter time (40 min), without stopping to replace or charge the battery. This result suggested that EPSO-based flight mission planning will create an optimal path for a VTOL drone operated with LoRaWAN^®^ to collect data from a large-scale rural livestock farm.

The demand of the modern agricultural and livestock industries for utilizing recent advanced technologies is rapidly growing. The convergence of agriculture and technology has provided many new entrepreneurial and investment opportunities. Hence, in the last decade, new companies have emerged to provide technology services to farmers, such as leasing agricultural drones for pesticide spraying, weed mapping, and crop status monitoring. In this regard, the system proposed in this article can be run by either experienced farm technicians or drone service provider companies.

## 5. Conclusions

In this paper, an aerial-based data collection system has been developed, which was based on the integration of the IoT, LoRaWAN^®^, and UAVs. The developed system consists of three main parts: (i) sensing nodes which are distributed throughout the farm which can be any kind of sensor node (beacon and/or unknown), (ii) a LoRaWAN^®^-based communication network, which collects data from sensors and conveys them to the cloud, and (iii) a path planning optimization technique, which optimizes the drone path for effectively sweeping the farm and collecting data from all established sensors. Based on the results, LoRaWAN^®^ is a reliable system capable of establishing a WSN over a large-scale farm. Furthermore, by integrating LoRaWAN^®^ into a drone, greater coverage can be achieved and it has robust performance with a maximum drone speed of 95 km/h. It was also shown that by increasing the flight altitude (e.g., 100 m~150 m), better LoRa coverage can be provided. Finally, the utilization of drones for aerial data collection not only can improve the availability of data, but can also accelerate farm monitoring procedures and enhance the productivity of farms, for example, by increasing production and optimizing the use of resources. In this regard, we believe that the developed system is a successful development in drone-based aerial data collection systems that provides a solution to tackle critical problems in farm management, aerial livestock monitoring, and collecting data from various IoT sensors.

## Figures and Tables

**Table 1:** Comparison of LPWAN wireless technologies.

**Description**	LoRa	SigFox	NB-IoT
**Coverage**	Urban: 5 km Rural: 20 km	Urban: 10 km Rural: 40 km	Urban: 1 km Rural: 10 km
**Frequency Band**	Unlicensed	Unlicensed	Licensed LTE Bands
**Maximum Data Rate**	50 kbps	100 bps	200 kbps
**Battery Life**	20 years	10 years	10 years
**Standardization**	LoRa Alliance	SigFox and ETSI	3GPP

**Table 3:** Technical specification of the LoRa multi-channel gateway.

Item	Specification
Microcontroller	Raspberry Pi 3B
LoRa Chipset	Sx1301
Frequency	915 MHz
Input	5 V–2.5 A
Antenna	SMA antenna 915 Mhz 50 ohm 5 dBi
RX Sensitivity	Min: −137 dBm

**Table 4:** Considered PL models’ accuracy evaluation metrics.

Model	MSE	RMSE	MAE	MAAPE	R	R2
FSPL	440.58	20.99	20.77	16.63	0.92	−9.25
ITM	223.23	14.94	14.65	11.71	0.92	−4.19
Fine-Tuned ITM	8.47	2.91	2.24	1.79	0.92	0.81
ECC-33	620.32	24.91	24.72	19.59	0.92	−13.43

**Table 5:** Cloud-RF^®^ simulation parameters.

Parameters	Value
Tx power	20 dBm
$G_{\mathrm{Tx}}$ (EN antenna gain) in dBi	2
$G_{\mathrm{Rx}}$ (GW antenna gain) in dBi	5
f in MHz	915
EN height AGL in m	1, 3, and 5
Rx sensitivity	−137 dBm
GW height (drone) AGL in m	0.1 (on the ground), 50, 100, and 150
Simulation radius	7 km

**Table 6:** Comparison of total traveling distance, operating hours, and the number of charging stops needed for different types of flight mission planning on the considered livestock farm.

Flight Mission Mode	Total Path Length (km)	No. of Stops/Battery Replacements	Continuous Operation Time (hours:minutes)
Square	212	2	02:15
Circular	175	1	01:50
PSO-based	79	0	00:50
ESPO-based	49	0	00:40

## Data Availability

The data presented in this study are available on request from the corresponding author.
